# Supplementary material for: Irreversible accumulated SERS behavior of the molecule-linked silver and silver-doped titanium dioxide hybrid system
Source: Nat Commun. 2020 Apr 14;11:1785. doi: 10.1038/s41467-020-15484-6 (PMC7156739; doi:10.1038/s41467-020-15484-6)
Supplement: Supplementary file 1 — Supplementary Information [file 41467_2020_15484_MOESM1_ESM.pdf]

Supplementary Information

Irreversible accumulated SERS behavior of molecule-linked silver and silver-doped titanium dioxide hybrid system

Zhou *et al.*

Supplementary Note 1: Substrates characterizations

Supplementary Figure 1a-g give the SEM images of Ag/Ag-doped TiO_2 substrates prepared with different concentrations of $AgNO_3$. Note that, when the concentrations of $AgNO_3$ increase to 0.4 mM, the morphology of as-prepared nanostructures were no obvious change. However, continually increase the concentration of $AgNO_3$ to 0.6 and 0.7 mM, some Ag nanoparticles were emerged on the surface of Ag/Ag-doped TiO_2 substrate. And UV-vis absorption spectra of as-synthesized of pure TiO_2 and Ag/Ag-doped TiO_2 substrates prepared with different concentrations of $AgNO_3$ is shown in Supplementary Figure 1h. For pure TiO_2, the wide absorption band below 400 nm corresponds to band energy of 3.2 eV. As compared with the pure TiO_2, the Ag/Ag-doped TiO_2 samples prepared with low concentrations of $AgNO_3$ (≤0.5 mM) exhibited a red shift of absorbance edge and a significant enhancement of absorption in the region 400-700 nm.[1] This is due to the localized surface plasmin resonance absorption of surface-deposited metallic Ag, and meanwhile, the absorption band between 400 and 650 nm shows an asymmetrical strong absorption behavior, which should be attributed to the optical absorption resulted by the doped energy level of Ag in the band gap of TiO_2, similar to the optical absorption of Ag_2O reported in the literature[2]. Moreover, the Ag/Ag-doped TiO_2 samples synthesized with high concentrations of $AgNO_3$ (> 0.5 mM), the absorption band approaches that of pure Ag along with a dramatically red-shift and broaden due to Ag nanoparticles emerged in the surface of Ag/Ag-doped TiO_2 substrate.

In addition, the X-ray diffraction (XRD) patterns of the TiO_2 and Ag/Ag-doped TiO_2 substrates prepared with 0.1, 0.3, 0.5, 0.6 and 0.7 mM of $AgNO_3$ are shown in Supplementary Figure 2a. It demonstrates that the diffraction peaks at 25.33°, 37.8°, 48.12°, 53.86°, 55.02° and

62.64° are assigned to the (101), (004), (200), (105), (211) and (204) planes of the anatase phase TiO_2 (PDF#21-1272). When the concentrations of $AgNO_3$ increase to 0.5 mM, the new diffraction peaks at 44.27°, 64.43° and 77.47° are assigned to the (200), (220) and (311) planes of Ag nanoparticle with face-centered-cubic structure (PDF#04-0783), indicating that the presence of a metallic Ag. And the other diffraction peaks at 40.17°, 53.02° and 70.65° are belonged to the (101), (102) and (103) planes of the Ti foil (PDF#44-1294). Supplementary Figure 2b gives the magnified XRD patterns of the TiO_2 and Ag/Ag-doped TiO_2 substrates prepared with 0.1, 0.3, 0.5, 0.6 and 0.7 mM of $AgNO_3$ in the 2θ range of 24-27°. It shows that the diffraction peak intensity at 25.33° of Ag/Ag-doped TiO_2 substrate decreased with increasing the concentrations of $AgNO_3$ as compared to that of the pure TiO_2 sample, which is possible due to the fact that Ag^+ ions, deposited on the surface of TiO_2 samples, suppressed the crystallization of the TiO_2 anatase phase[3,4]. In addition, the anatase (101) peaks of TiO_2 in the Ag/Ag-doped TiO_2 substrates slight shifted to a smaller diffraction angle, indicating that the Ag/Ag-doped TiO_2 nanostructure occurs a lattice distortion. It should be attributed to the diffusion and rearrangement of the Ti^{4+} and O^{2-} ions in the anatase TiO_2 and the disturbed by the Ag^+ ions spreading into the anatase TiO_2, leading to distortion in the crystal lattice of TiO_2[3,5]. In spite of fact that the radius of Ag^+ ion (126 Å) is larger than that of Ti^{4+} ion (68 Å), the Ag^+ ion could still enter into the crystal lattice of TiO_2 to replace Ti^{4+} ion by the sol-hydrothermal process, induced O vacancies or deficiencies of Ti^{4+}, which results in a shifting to small angle, peak broadening and decline[4,5].

Furthermore, X-ray photoelectron spectroscopy (XPS) analysis is performed for Ag-TiO_2, TiO_2 and Ag/Ag-doped TiO_2 substrates prepared with 0.1, 0.3, 0.5 and 0.7 mM of $AgNO_3$, as

shown in Supplementary Figure 3. The fully scanned spectra show that Ti, O and C elements exist in the pure TiO_2, whereas, Ti, O, Ag and C elements exist in the Ag-TiO_2 and Ag/Ag-doped TiO_2 substrates, in which no traces of any other impurity were observed, except for the adventitious carbon from ambient environment. To obtained further evidence about the interaction between the Ag and TiO_2, the high resolution XPS spectra of Ag 3d, Ti 2p and O 1s are displayed in Supplementary Figure 3b–d. The Ag $3d_{5/2}$ and Ag $3d_{3/2}$ peaks in the Ag-TiO_2 sample appeared at 368.2 and 374.2 eV of binding energies, respectively. This typical spin energy splitting of the 3d doublet (6 eV) proves that Ag certainly presents in the Ag-TiO_2 sample in the form of metallic Ag[6]. And there is no Ag 3d peak in the TiO_2 precursor due to the absence of Ag element. As compared to Ag-TiO_2, Ag 3d peaks in the Ag/Ag-doped TiO_2 samples showed widening and shifting to lower binding energy, suggesting the existence of silver oxidation states. The Ag 3d peaks in the Ag/Ag-doped TiO_2 substrates were fitted by using software program XPSPeak 4.1, indicating that the chemical states of Ag exist mainly as Ag^+ (oxide) and Ag^0 (metallic Ag). It is particularly noted that the chemical state of Ag in the Ag/Ag-doped TiO_2 sample prepared with 0.1 mM $AgNO_3$ are mainly Ag^+ (oxide), which is ascribed to Ag^+ replacing Ti^{4+} at TiO_2 lattice site. With increasing the concentration of $AgNO_3$, the area percentages of the chemical state of Ag^0 (metallic Ag) obviously increases. This is because the Ag^+ doping content reach saturation and the excess silver is deposited on the TiO_2 in the form of metallic Ag. Thus, the prepared samples not only have doping of Ag^+ but also deposition of metallic Ag. Besides, the Ti 2p peaks at 458.2 and 464.0 eV correspond to Ti $2p_{3/2}$ and Ti $2p_{1/2}$, respectively[7,8]. For pure TiO_2, the peak sitting between Ti $2p_{3/2}$ and Ti $2p_{1/2}$ lines is about 5.8 eV, suggesting the existence of the Ti^{4+} oxidation state[8]. And the peak

positions of Ti 2p of Ag-TiO_2 show a positive shift than that of pure TiO_2, indicating a lower electron cloud density of the Ti atoms in the Ag-TiO_2 sample[9,10]. It means that the Fermi level of Ag is lower than the conduction band of TiO_2 so that the electron transfer can occur between TiO_2 and the Ag deposited on the surface of TiO_2[11,12]. However, compared to pure TiO_2, the Ti 2p peaks in the Ag/Ag-doped TiO_2 samples shifted into lower binding energy. By fitting XPS spectra, Ti 2p peaks of the Ag/Ag-doped TiO_2 samples can be divided into Ti^{3+} and Ti^{4+} peaks. During the sol-hydrothermal process, Ag^+ could disturb the formation of TiO_2, that is, Ag^+ could replace Ti^{4+} at TiO_2 lattice site, resulting in some changes of Ti^{4+} into Ti^{3+}. Thus, the shifting of Ti 2p peak to lower binding energy is attributed to Ag^+ doping into the TiO_2 so that the radius of Ti ion is expand and the electron moves far from the Ti nuclei[13]. In addition, O 1s peak at 529.4 eV is attributed to signal of oxygen in TiO_2 lattice. Compared to that of Ag-TiO_2 and bare TiO_2, O 1s peaks of the Ag/Ag-doped TiO_2 samples shifted into lower binding energy, which indicates the decrease of the coordination number of Ti and the lengthen of the Ti-O bond[3]. This result also supports that the presence of Ti^{4+} is decreased.

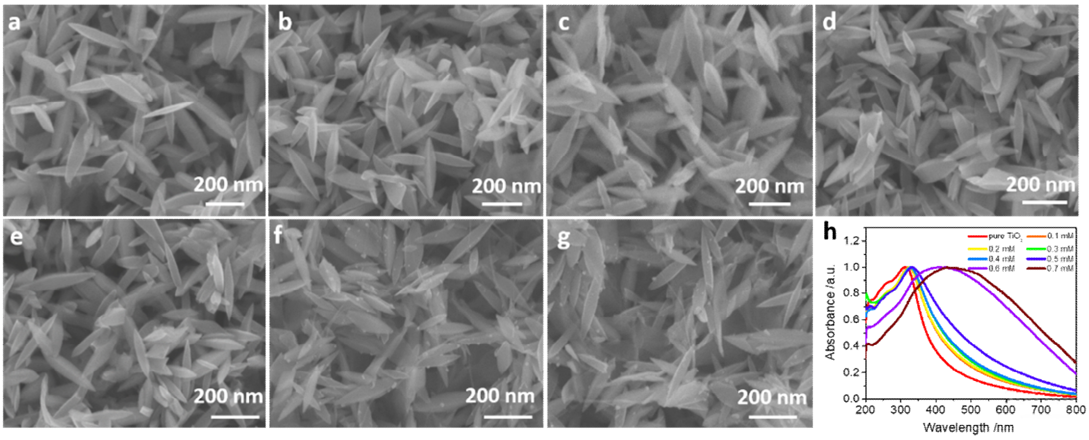

Supplementary Figure 1 **Characteristics of Ag/Ag-doped TiO_2 substrates.** SEM images of Ag/Ag-doped TiO_2 substrates prepared with different concentrations of $AgNO_3$: (a) 0, (b) 0.1, (c) 0.2, (d) 0.3, (e) 0.4, (f) 0.6 and (g) 0.7 mM. (h) UV-vis absorption spectra of as-synthesized of pure TiO_2 and Ag/Ag-doped TiO_2 substrates prepared with different concentrations of $AgNO_3$.

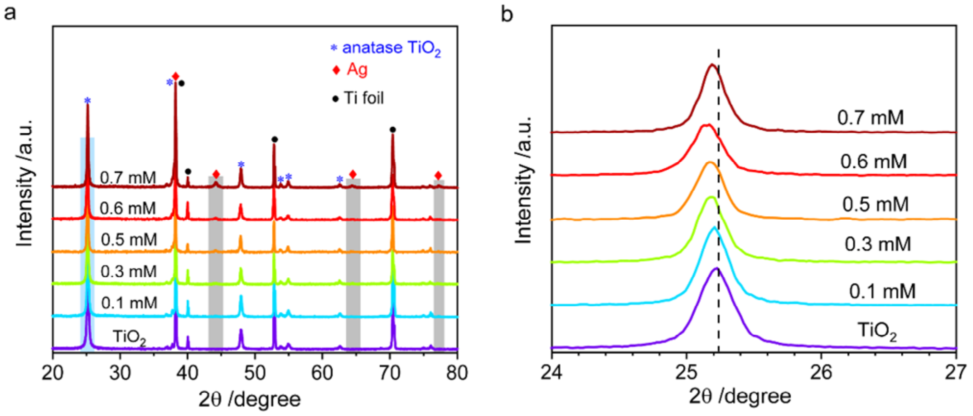

Supplementary Figure 2 **X-ray diffraction analysis.** (a) XRD patterns of the TiO_2 and Ag/Ag-doped TiO_2 substrates prepared with 0.1, 0.3, 0.5, 0.6 and 0.7 mM of $AgNO_3$ and (b) the corresponding magnified XRD patterns in the 2θ range of 24-27º.

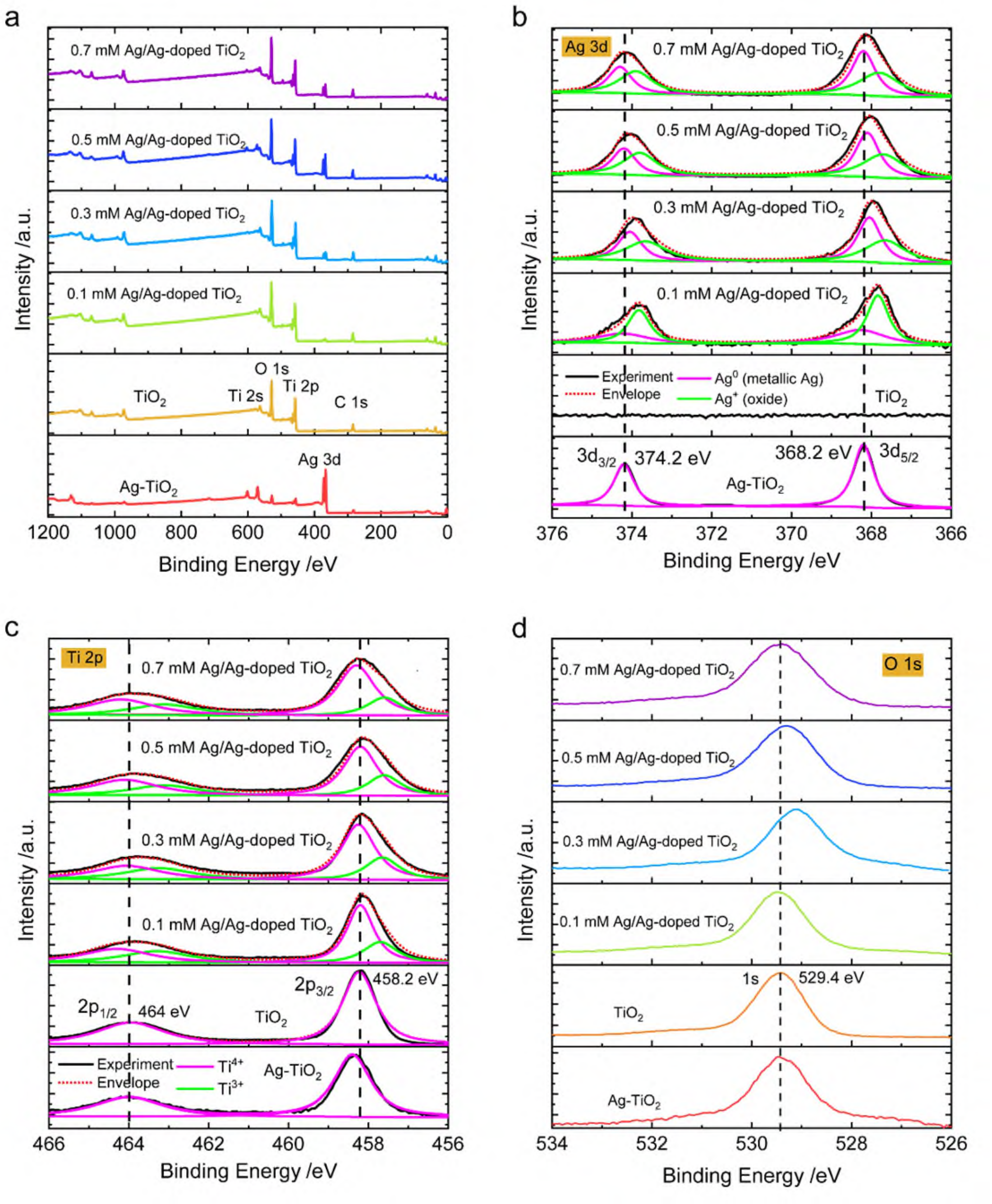

Supplementary Figure 3 X-ray photoelectron spectroscopy (XPS). (a) XPS survey spectra of Ag deposition on the surface of TiO_2 (Ag-TiO_2) by magnetron sputtering, TiO_2 and Ag/Ag-doped TiO_2 substrates prepared with 0.1, 0.3, 0.5 and 0.7 mM of $AgNO_3$. And the high-resolution XPS spectra of (b) Ag 3d, (c) Ti 2p and (d) O 1s show the binding energies.

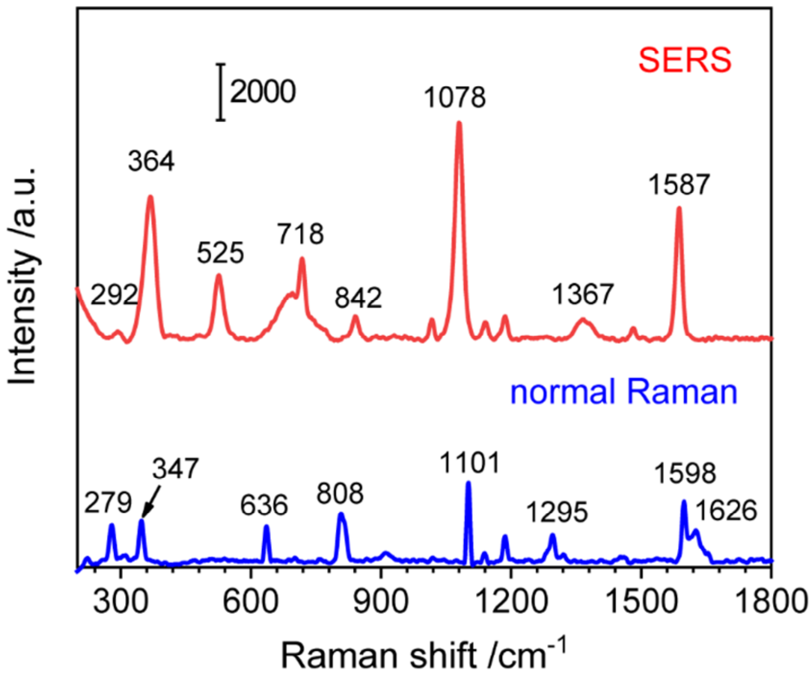

Supplementary Figure 4 SERS measurement. SERS spectrum (red line) of 4MBA adsorbed on the Ag/Ag-doped TiO_2 substrate prepared with 0.5 mM $AgNO_3$ and the normal Raman spectrum (blue line) of 4MBA powder.

In Supplementary Figure 4, the SERS spectrum of 4MBA adsorbed on the Ag/Ag-doped TiO_2 substrate is consistent with that of the previously reported for 4MBA adsorbed on Ag-TiO_2 nanoparticles[14]. It can be found that at the same excitation wavelength, SERS spectrum of 4MBA is very similar to its normal Raman spectrum. But, the characteristic SERS peaks of 4MBA are observed to display slightly different from those of the normal Raman spectrum in frequency and intensity, which can be attributed to the different adsorption state of 4MBA molecule and the intrinsic properties of electromagnetic and chemical enhancement. Therefore, in our work, the reported spectra are the SERS spectra rather than normal Raman spectra.

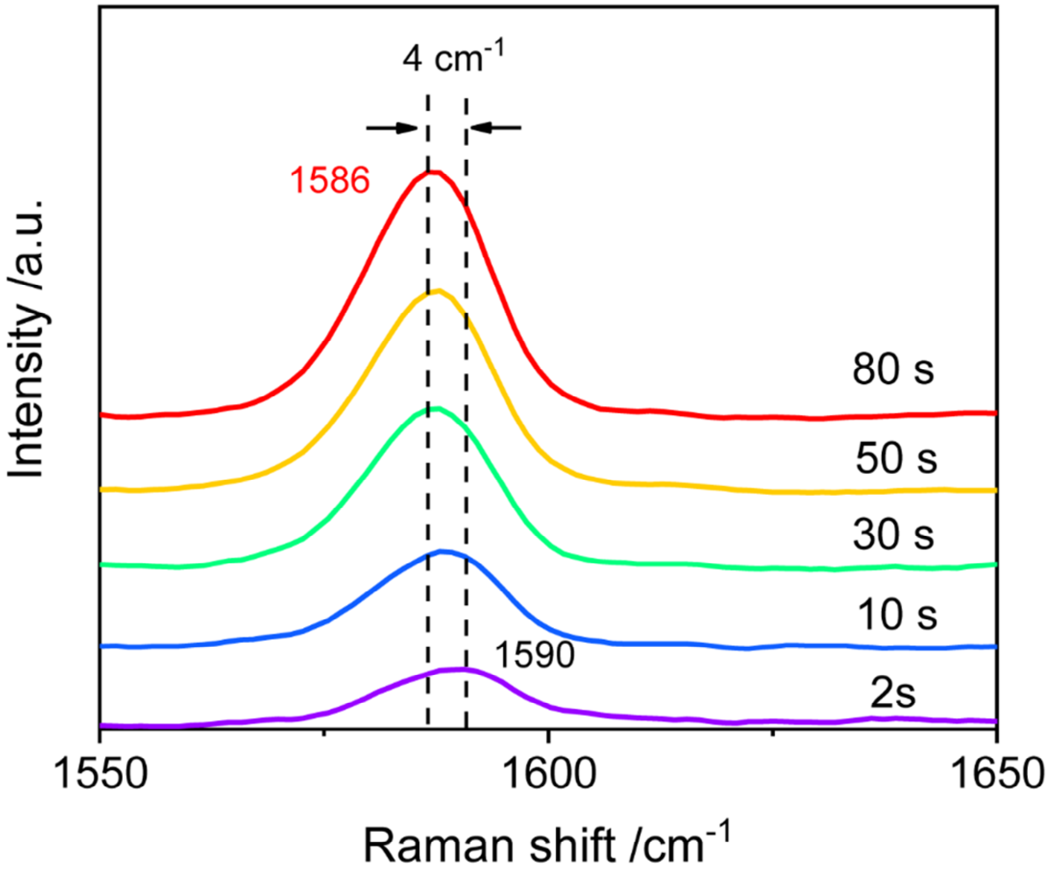

Supplementary Figure 5 Raman frequency shifting. Magnified irradiation time-dependent SERS spectra between 1550 and 1650 cm^{-1} for the 4MBA/Ag/Ag-doped TiO_2 hybrid system prepared with 0.5 mM $AgNO_3$.

From Supplementary Figure 5, it can be seen that with increasing of irradiation time, accompanying the reorientation of 4MBA molecules to the Ag/Ag-doped TiO_2 substrate, the frequency of aromatic ring vibration mode experiences a red-shift from 1590 to 1586 cm^{-1}, because the changes of bonding interaction, bond length and bond angle are correlated with the adsorption geometries on the surface of substrate[15,16].

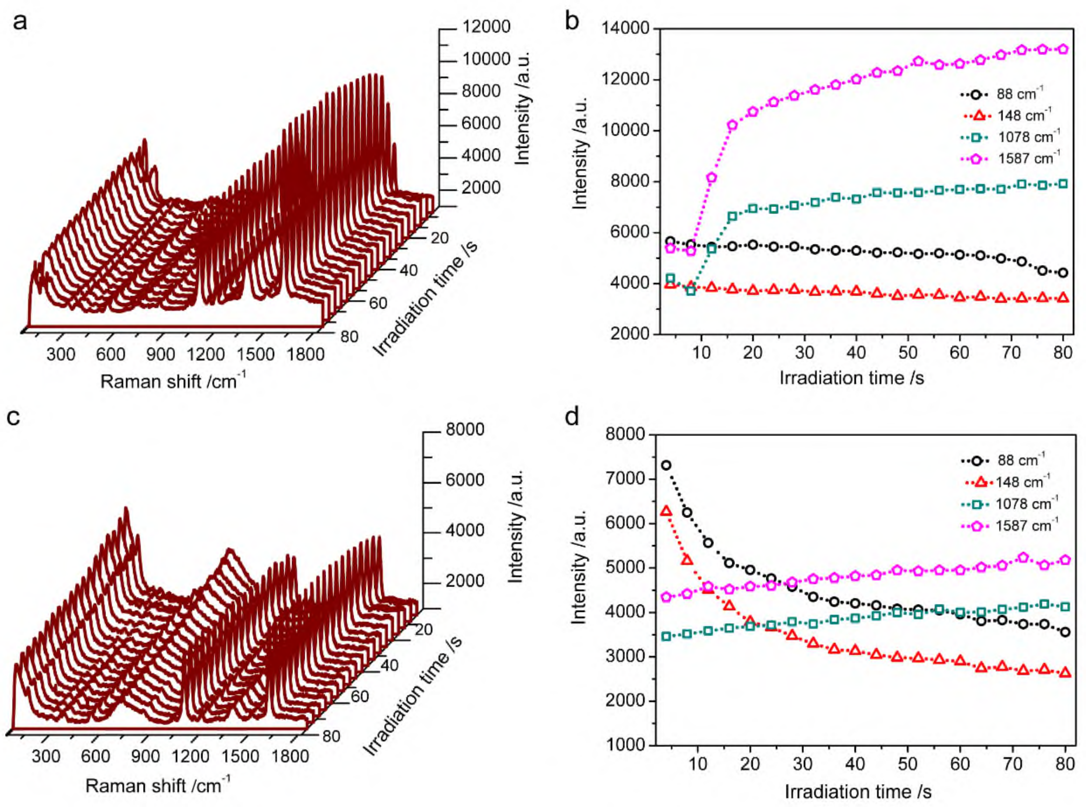

Supplementary Figure 6 SERS spectra under different exciting wavelengths. Irradiation time-dependent SERS spectra of the 4MBA/Ag/Ag-doped TiO_2 hybrid system prepared with 0.5 mM $AgNO_3$ under exciting of (a) 532 and (c) 633 nm laser with the power of 1.2 mW and 1.7 mW, respectively; (b and d) the corresponding SERS intensities of the peaks at 88, 148, 1078 and 1587 cm^{-1}.

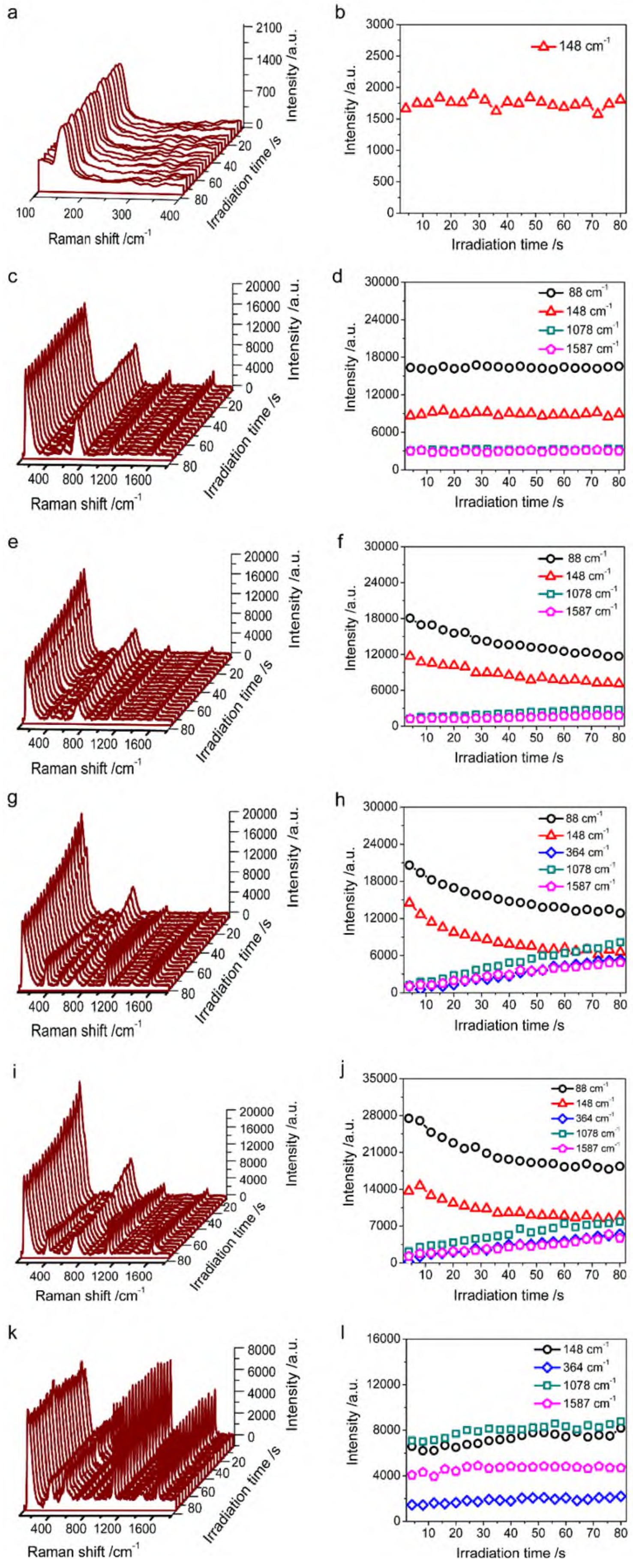
a
b
c
d
e
f
g
h
i
j
k
l
Raman shift /cm-1
Irradiation time /s
Intensity /a.u.
88 cm-1
148 cm-1
364 cm-1
1078 cm-1
1587 cm-1

Supplementary Figure 7 SERS spectra of different samples. Irradiation time-dependent SERS spectra of the 4MBA/Ag/Ag-doped TiO_2 hybrid systems prepared with the different concentrations of $AgNO_3$ of (a) 0, (c) 0.1, (e) 0.3, (g) 0.4 and (i) 0.7 mM, and their temporal evolutions of SERS peak intensities (b, d, f, h, and j). (k) The irradiation time-dependent SERS spectra of Ag-4MBA and (l) its temporal evolutions of SERS peak intensities.

As shown in Supplementary Figure 7, in the case of low concentration $AgNO_3$ (0 and 0.1 mM), the main SERS signals of Ag, TiO_2 and 4MBA maintain invariant with increasing the irradiation time due to no or few Ag was incorporated into TiO_2 nanostructure. Then, continually increasing the concentration of $AgNO_3$, the main SERS signals of 4MBA show exponential increase trends, while the SERS intensity of Ag and TiO_2 present reverse trends. For the case of only Ag nanoparticles, the main SERS signals of Ag and 4MBA are also basically maintain invariant with increasing the irradiation time. This further illustrate that the irradiation-time dependent SERS behaviors of 4MBA/Ag/Ag-doped TiO_2 hybrids system are ascribed to the synergistic effect of Ag and TiO_2.

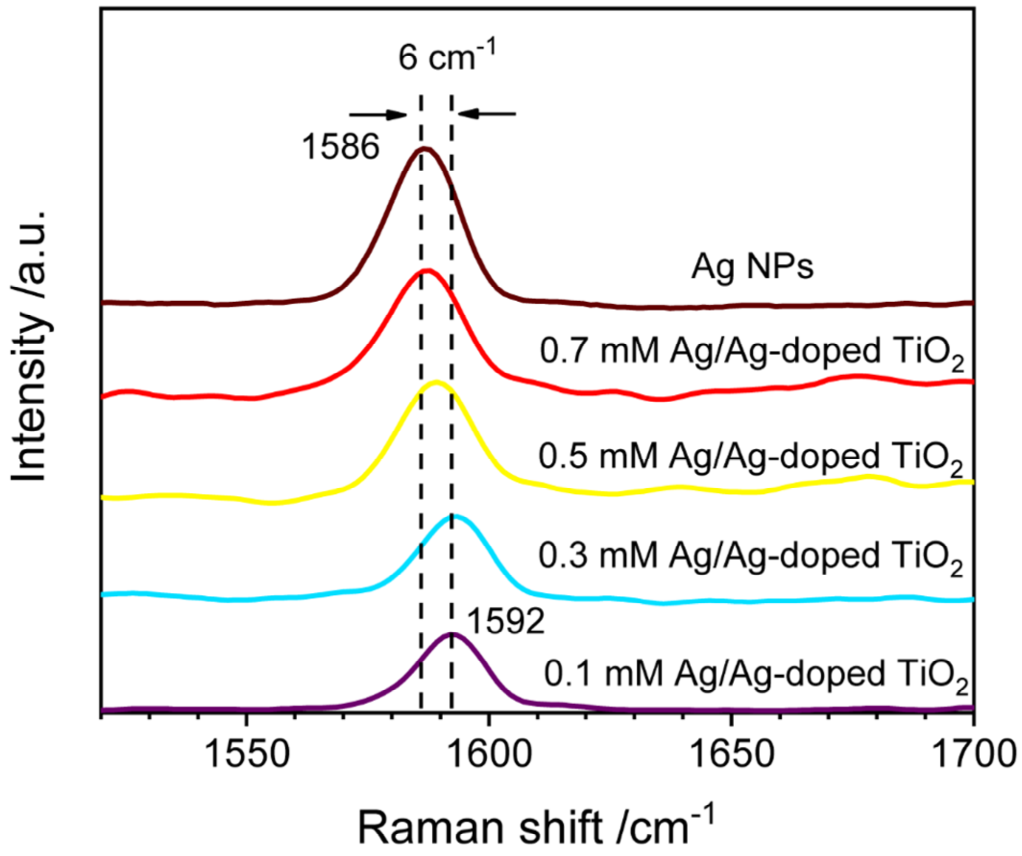

Supplementary Figure 8 SERS spectra of different samples. Magnified SERS spectra of 4MBA adsorbed on the Ag NPs and the Ag/Ag-doped TiO_2 substrates prepared with different concentrations of $AgNO_3$.

In Supplementary Figure 8, with increase of the concentration of $AgNO_3$ aqueous solution, the frequency of the Raman mode assigned to the aromatic ring characteristic vibration shifts from 1592 to 1587 cm^{-1}, very close to the Raman mode at 1586 cm^{-1} in the case of Ag NPs, which can be ascribed to the contribution of electromagnetic enhancement of surface-deposited Ag NPs due to the increase of the loading amount and aggregation degree of Ag NPs on the Ag/Ag-doped TiO_2 substrate[14].

Supplementary Note 2: Calculation of SERS enhancement factor

To evaluate the plasmonic properties of as-prepared Ag/Ag-doped TiO_2 substrates, the enhancement factors (EFs) are calculated by using the following equation[17]

$$\mathrm{EF} = (I_{\mathrm{SERS}}/I_{\mathrm{bulk}}) \times (N_{\mathrm{bulk}}/N_{\mathrm{SERS}}) \qquad (1)$$

where I_{SERS} is the integrated intensity of a SERS mode such as the ring-breathing mode (υ(C–S)) at 1078 cm^{-1}, I_{bulk} is the intensity of the same mode in the Raman spectrum of 4MBA, N_{SERS} and N_{bulk} are the numbers of 4MBA molecules adsorbed on the SERS substrate and the number of 4MBA molecules in powder at the laser spot, respectively. The diameter of the laser spot was calculated to be 2.4 μm using the equation[18]: $D_{\mathrm{dimater}} = (\lambda/NA) \times 1.22$, in which λ and NA are 785 nm and 0.4 for the specifications of Raman spectrometer, respectively. N_{SERS} was calculated to be 1.36×10^7 based on the following equation: $N_{\mathrm{SERS}} = (N_{\mathrm{A}} \times A)/\sigma$, in which N_A is Avogadro constant, A is effective area occupied by 4MBA molecules under the laser irradiation, and σ=2.0 × 10^9 cm^2 mol^{-1} is the per mol area of self-assembled monolayer of 4MBA molecules[19]. And N_{bulk} is calculated to be 7.1 × 10^{10} with using 1.5 g·cm^{-3} of 4MBA molecular density and 154.19 g·mol^{-1} of molecular weight. Supplementary Figure 9 give the SERS spectra of 4MBA adsorbed on the Ag/Ag-doped TiO_2 substrates before and after NIR irradiation and the normal Raman signal of 4MBA powder at the same measure condition. And the values of I_{SERS} and I_{bulk} of 4MBA before and after NIR irradiation can be obtained from Supplementary Figure 9, respectively. As a result, the EFs of each Ag/Ag-doped TiO_2 substrate is listed in Supplementary Table 1. It can be found that the values of EF are increased with increasing the concentration of Ag NO_3 before NIR irradiation, which were caused by the electromagnetic enhancement. And after NIR irradiation, the values of EF also show increases, this is because of the molecular reorientation. Of particular note is the Ag/Ag-doped TiO_2 substrate prepared with 0.5 mM $AgNO_3$ display well SERS activities, especially after NIR irradiation, the EF increased from 2.88 × 10^5 to 1.68×10^6, nearly six-fold, which is better than

that of bare-Ag NPs. In addition, we also compared the EF of the prepared Ag/Ag-doped TiO_2 substrate to the literature reported values, as listed in Supplementary Table 2. It clearly shows that the 4MBA/Ag/Ag-doped TiO_2 hybrid system after NIR irradiation achieves a better SERS enhancement. Therefore, the prepared Ag/Ag-doped TiO_2 substrate will provide new opportunities in trace detection applications.

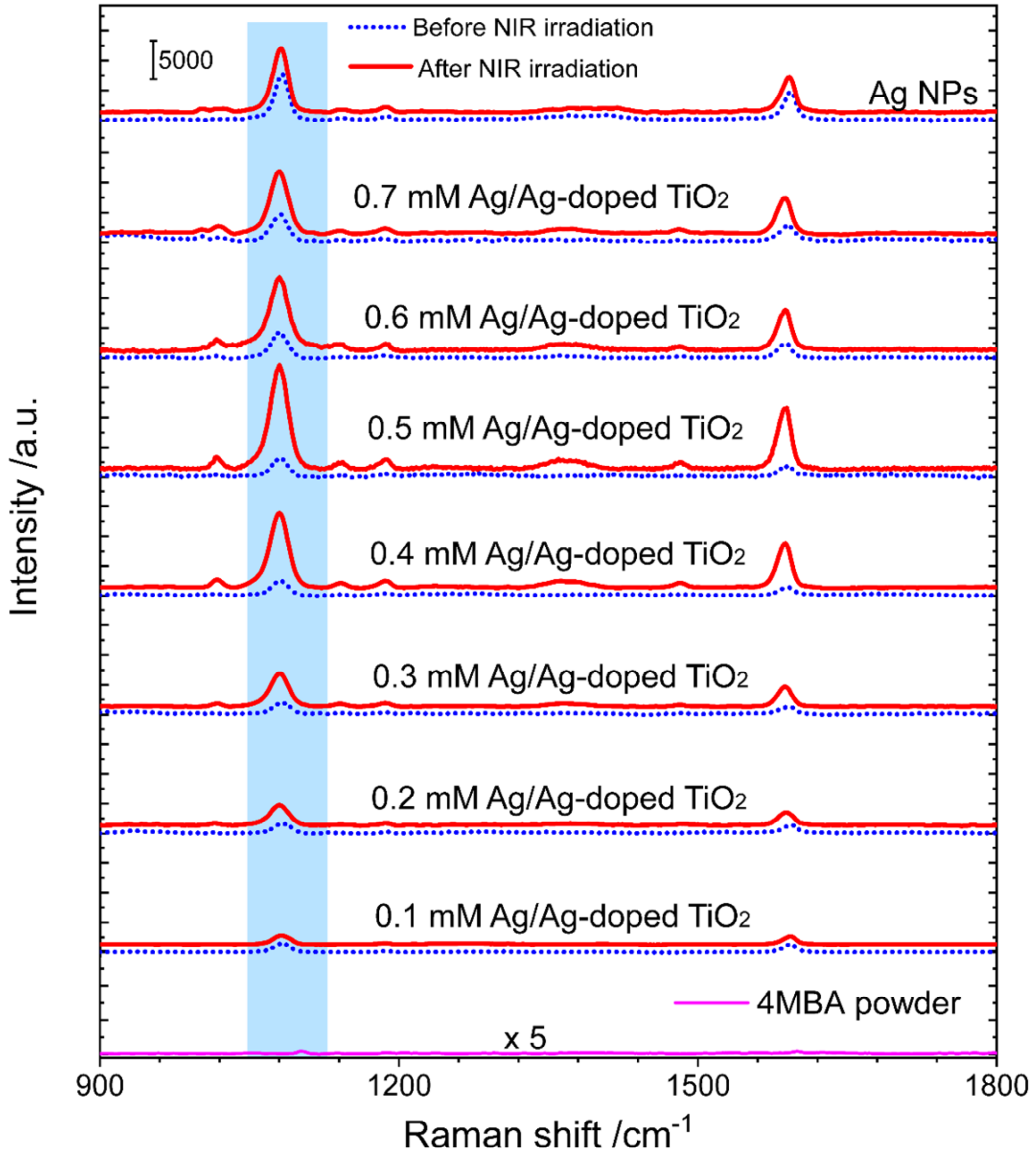

Supplementary Figure 9 **SERS enhancement**. SERS spectra of 4MBA-Ag NPs and each 4MBA/Ag/Ag-doped TiO_2 hybrid systems before and after NIR irradiation (100 s) and the normal Raman signal of 4MBA powder at the same measure conditions.

Supplementary Table 1

SERS enhancement factors of the prepared substrates and Ag NPs

**Substrate**	**Ag/Ag-doped TiO_2**							**Ag NPs**
$AgNO_3$	0.1 mM	0.2 mM	0.3 mM	0.4 mM	0.5 mM	0.6 mM	0.7 mM	
$EF_{(1)}$	1.37×10^5	1.67×10^5	2.1×10^5	2.31×10^5	2.88×10^5	3.71×10^5	4.24×10^5	6.1×10^5
$EF_{(2)}$	1.45×10^5	3.64×10^5	5.64×10^5	1.24×10^6	1.68×10^6	1.34×10^6	9.92×10^5	9.03×10^5

Note: (1) before NIR irradiation (2) after NIR irradiation

Supplementary Table 2 Comparison of enhancement factors

**SERS substrate**	**EF**	**Reporter molecule**	**Reference**
Ag decorated TiO_2 nanorod	$\sim3.1\times10^5$	rhodamine 6G	Fang et al.[20]
Au coated on TiO_2 spheres	1.4×10^5	rhodamine 6G	Li et al.[21]
Ag coated on TiO_2 Nanofibers	6.7×10^5	4-mercaptopyridine	Song et al.[22]
$Fe_3O_4/SiO_2/ZnO/Ag$	$\sim8.2\times10^5$	4-mercaptobenzoic acid	Han et al.[23]
Ag/TiO_2 scaffold	4.36×10^5	rhodamine 6G	Tan et al.[24]
ZnO/Ag, Ag/ZnO and ZnO/Ag/ZnO	7.4×10^6, 4.4×10^5 and 8.9×10^4	rhodamine 6G	Praveena et al.[25]
Au/ZnO/Si	$\sim10^5$	rhodamine 6G	Chan et al.[26]
Ag/ZnO nanorod	1.23×10^6	rhodamine 6G	Tao et al.[27]
ZnO nanorod-Ag	2.64×10^6	rhodamine 6G	Liu et al.[28]
Ag/Ag-doped TiO_2	1.68×10^6	4-mercaptobenzoic acid	This work

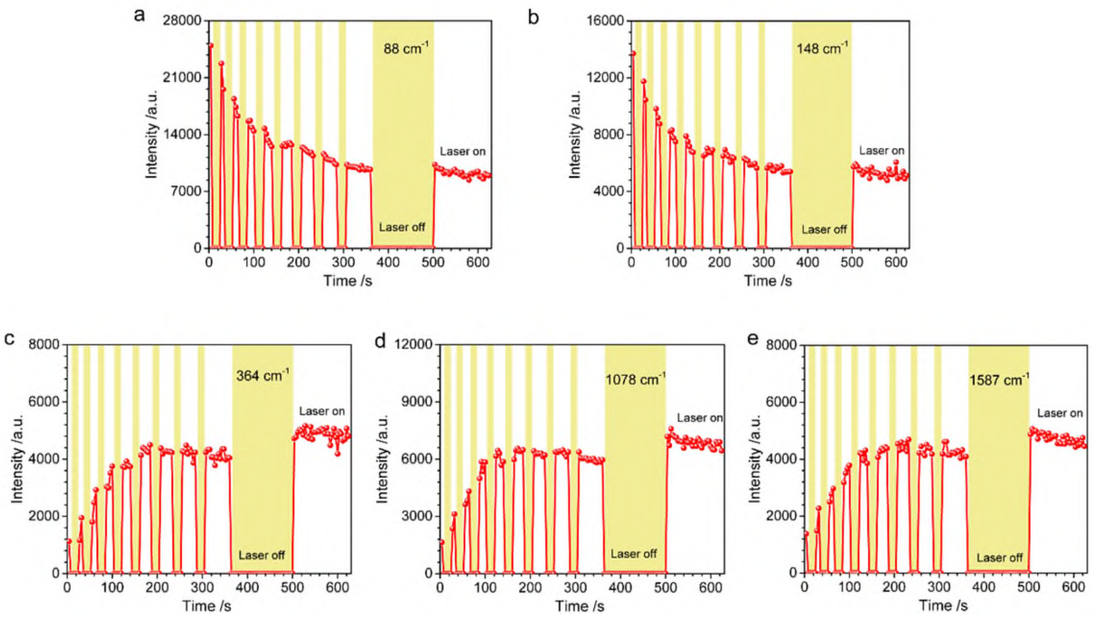

Supplementary Figure 10 Time-evolution of SERS peaks of sample. Irradiation time-dependent SERS intensity of 4MBA/Ag/Ag-doped TiO_2 hybrids system prepared with 0.5 mM $AgNO_3$ for the peaks of (a) 88 cm^{-1}, (b) 148 cm^{-1}, (c) 364 cm^{-1}, 1078 cm^{-1} and (d) 1587 cm^{-1}, under temporarily blocking and opening of the laser with wavelength of 785 nm.

Supplementary Note 3: Control experiments for temperature effect

Firstly, a synthesized Ag/Ag-doped TiO_2 substrate was cleaved into four pieces, then three of them were coated with 4MBA but the fourth without any coating, and namely sample A, B, C and D. Next, their irradiation time-dependent SERS spectra were measured as follow conditions. Sample A was kept at room temperature, sample B was heat and kept at 45 ºC, sample C was heat to 45 ºC and then cooled down to room temperature, sample D was heated to 45 ºC and then cooled down to room temperature before coated with 4MBA. As shown in Supplementary Figure 11a-c, the intensities of peaks at 1078, 88 and 148 cm^{-1} are irradiation time-dependent, respectively. The results of sample A and sample B confirm that the SERS intensity measured at higher temperature is stronger than that at room temperature. For sample C and sample D, the intensity of peak at 1078 cm^{-1} is like that of sample A at room temperature. This indicates that there is no change of the SERS spectra of 4MBA even after the adsorbed molecule and the substrates went through the heating and cooling step, which confirms that the temperature-induced SERS enhanced behavior is a reversible process for 4MBA. Based on the above temperature-correlated experiments, it is deduced that there is no change of the 4MBA molecule characteristics when it goes through the relatively high temperature stressing, thus the impact of temperature on the irreversible enhancement of the SERS signal of 4MBA can be excluded. However, the SERS behavior of Ag/Ag-doped TiO_2 substrate is different. The intrinsic Raman intensities of Ag and TiO_2 for the four samples are shown in Supplementary Figure 11b and c, respectively. For sample A the Raman signals of both Ag and TiO_2 decrease in an exponential behavior. For sample B there is negligible change. For sample C and sample D, their Raman spectra show higher intensities than that of sample A and

a similar decline trend with increasing irradiation time, although their decreasing rates are smaller than that of sample A. The above experiments confirm that the temperature induces a permanent change to the Ag/Ag-doped TiO_2 substrate, resulting in the discrepancy of the Raman behavior.

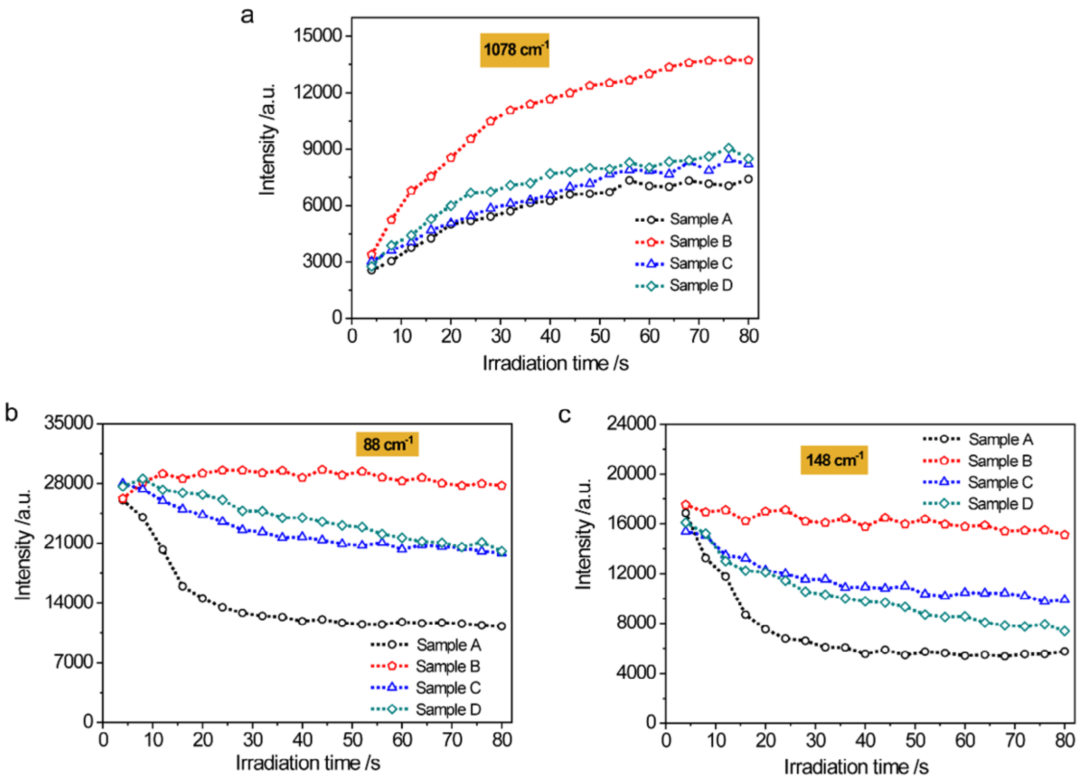

Supplementary Figure 11 Control experiments. Irradiation time-dependent SERS peak intensities of (a) 4MBA (1078 cm^{-1}), (b) Ag (88 cm^{-1}) and (c) TiO_2 (148 cm^{-1}) for the 4MBA/Ag/Ag-doped TiO_2 hybrids system prepared with 0.5 mM $AgNO_3$, with/without heating treatment. Herein, the irradiation time-dependent SERS intensities of peaks at 364 and 1587 cm^{-1} are not displayed due to their trends same as that of SERS peak at 1078 cm^{-1}.

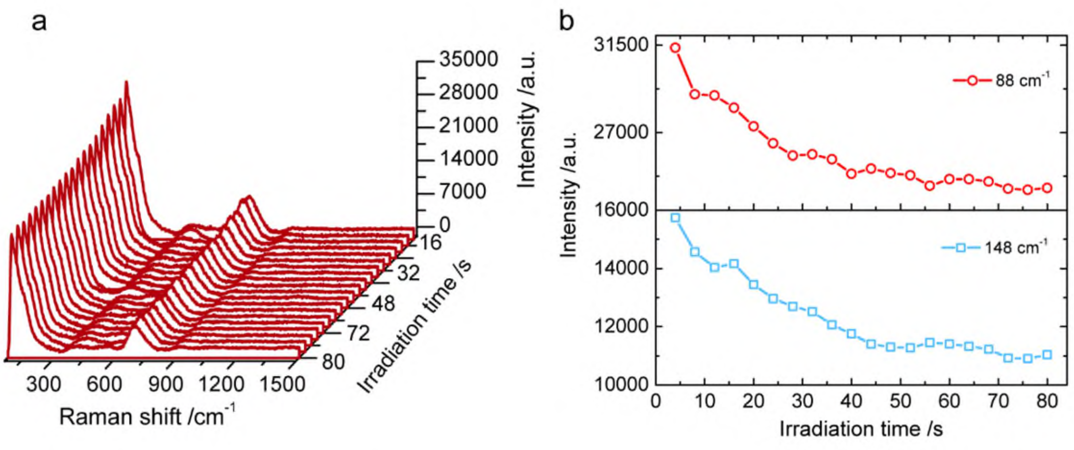

Supplementary Figure 12 SERS behavior of substrate. (a) Irradiation time-dependent SERS spectra of Ag/Ag-doped TiO_2 substrate prepared with 0.5 mM $AgNO_3$ without 4MBA; (b) Temporal evolution of Raman peak intensity at 88 and 148 cm^{-1}.

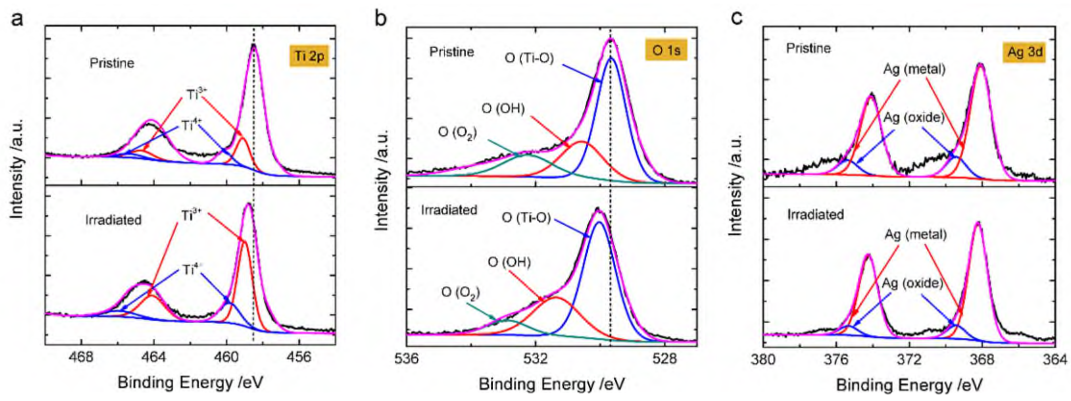

Supplementary Figure 13 High-resolution X-ray photoelectron spectroscopy (XPS). XPS spectra of the Ag/Ag-doped TiO_2 substrate prepared with 0.5 mM $AgNO_3$ before and after irradiation: (a) Ti 2p, (b) O 1 s and (c) Ag 3d.

As shown in Supplementary Figure 13, by fitting of high-resolution X-ray photoelectron spectroscopy, the XPS peaks of Ti 2p, O 1s and Ag 3d of Ag/Ag-doped TiO_2 substrate can be divided into different valence state peaks, respectively. After irradiation of 785 nm laser, it can be found that the respective area percentages of Ti^{3+} and Ti^{4+} obviously increases and the peaks of Ti 2p shift towards to higher binding energy comparing with that of pristine sample. It reveals that a lower electron cloud density of the Ag/Ag-doped TiO_2 substrate due to the remove of V_O defect after laser irradiation[8]. Moreover, the wide and asymmetrical spectra of O 1s can be further fitted into three peaks including crystal lattice oxygen O (Ti-O), surface hydroxyl groups O (OH) and adsorbed oxygen O (O_2)[29,30]. The respective area percentages of O (Ti-O) and O (OH) of Ag/Ag-doped TiO_2 substrate after exposure to 785 nm laser were higher than that of pristine samples and the peaks of O 1s shifts towards to higher position, which can be ascribed to the remove of the V_O defect causing the increase the oxygen species of O (Ti-O). While, the area percentage of O (O_2) was reduced due to the oxygen release from the surface of the samples under the irradiation of 785 nm laser. Furthermore, the chemical

state of Ag in Ag/Ag-doped TiO_2 substrate exist mainly as Ag (metal) and Ag (oxide)[31]. And after irradiation, the area percentage of the Ag (metal) increase, simultaneously, the area percentage of Ag (oxide) reduced. It can be attributed to the destroyed of the weak interactions between Ag ions and the production of elemental Ag under the laser irradiation. XPS data further support the fact that the irradiation reduces the density of V_O defects in the substrate and consequently improve the crystalline structure[32].

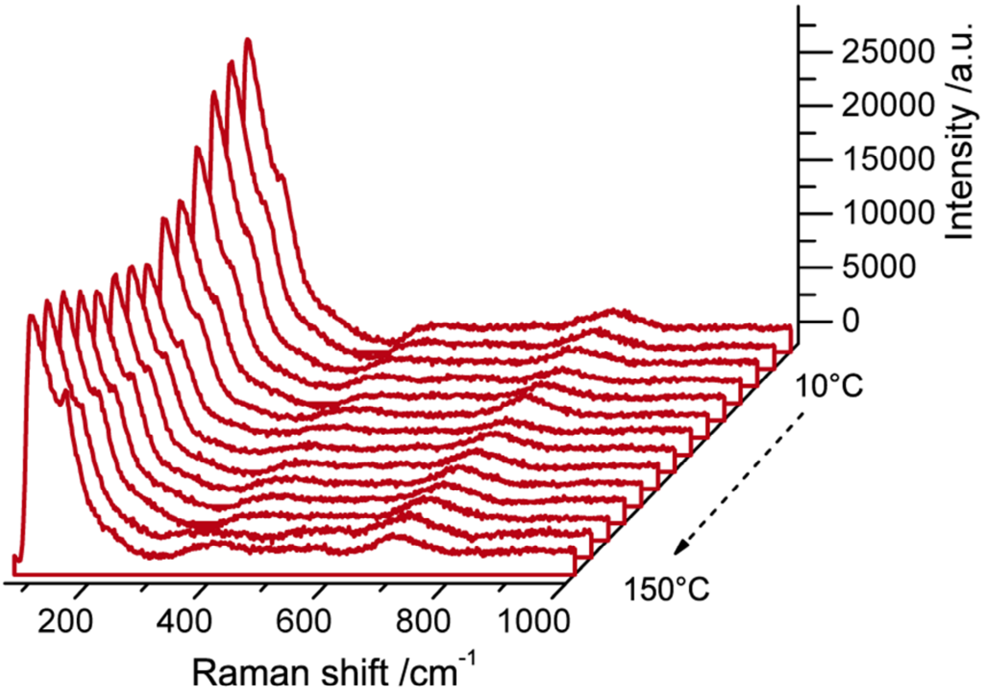

Supplementary Figure 14 **Temperature effect.** Temperature-dependent SERS spectrum of bare Ag/Ag-doped TiO_2 substrate prepared with 0.5 mM $AgNO_3$.

Supplementary Note 4: First principles calculation of Raman intensity for anatase TiO_2

As well known, the Raman intensity of a vibration mode can be expressed as[33,34,35]:

$$\mathrm{I} \propto N \times \tilde{\alpha}^2 \qquad (2),$$

where N is the total number of certain vibration mode in the system, $\tilde{\alpha}$ is the polarizability tensor invariant scalar given by:

$$\tilde{\alpha}^2 = \frac{45\bar{\alpha}^2 + 7\bar{\gamma}^2}{45} \qquad (3),$$

where $\bar{\alpha}$ is the average invariant of the polarizability tensor or also called the 'reduced trace of the matrix', and defined as:

$$\bar{\alpha} = \frac{1}{3}\left[\alpha_{xx} + \alpha_{yy} + \alpha_{zz}\right] \qquad (4),$$

$\bar{\gamma}$ is the polarizability tensor directivity invariant or also known as the 'anisotropy' parameter of the matrix, and follows the formula:

$$\bar{\gamma}^2 = \frac{1}{2}\left[\left(\alpha_{xx} - \alpha_{yy}\right)^2 + \left(\alpha_{yy} - \alpha_{zz}\right)^2 + \left(\alpha_{zz} - \alpha_{xx}\right)^2\right] + 3\left[\alpha_{xy}^2 + \alpha_{xz}^2 + \alpha_{yz}^2\right] \quad (5),$$

where $\alpha_{xx}, \alpha_{yy}, \alpha_{zz}, \alpha_{xy}, \alpha_{xz}$, and α_{yz} are the polarizability tensor elements. These invariants are independent of the coordinate frame in which the matrix representation is given, i.e. independent of the orientation of the molecule.

To understand the Raman characteristics of anatase TiO_2, we studied the correlation between the polarizability tensor of the anatase TiO_2 and the concentration of V_O defects by using density functional theory (DFT) calculations. As the material polarizability is always closely related to the atomic structure, for simplifying the calculations without loss of generality, a supercell of anatase TiO_2 crystal with 300 atoms was employed in our simulation. The ultra-soft pseudo-potential and plane-wave expansions of wave functions and potentials are

implemented during the calculation[36]. The exchange correlation energies were treated within the generalized gradient approximation according to the literature[37]. Before the calculations, the cut-off energy and points were tested. For structure optimization, the conjugate gradient method was used, and the ion positions were optimized until the residual force was less than 0.01 eV/ Å. To introduce different concentrations of the V_O defect in the anatase TiO_2 structure, specific number (M) of oxygen atoms in the supercell was removed, and thus the V_O defect concentration of the created supercell was M/300. The polarizabilities of anatase TiO_2 with different V_O defects were obtained according to the anisotropic Clausius-Mossotti formula by calculating the dielectric tensor[38-40]. And the calculated polarizability tensor of the anatase TiO_2 with different V_O defect concentrations were listed in Supplementary Table 3. As shown in Figure 5a of main text, a polynomial function was used to fit these data and the corresponding fitting curve was plotted by the following equation:

$$\tilde{\alpha}^2 = 2.2571 \times 10^{-11} - 1.9577 \times 10^{-9}x + 9.6547 \times 10^{-7}x^2$$
$$-4.904 \times 10^{-5}x^3 \qquad (6)$$

where x is the concentration of V_O defects. It is obviously seen that the polarizability of the anatase TiO_2 increases with the growth of V_O defect concentrations, except for very low and high concentrations of V_O defect.

Supplementary Table 3 **Calculated results**

x (%)	$\bar{\alpha}$	$\bar{\gamma}^2$	$\tilde{\alpha}^2$
0	$4.73694\text{x}10^{-6}$	$3.58663\text{x}10^{-14}$	$2.24441\text{x}10^{-11}$
0.333	5.04159×10^{-6}	3.10972×10^{-13}	2.5466×10^{-11}
0.667	6.04791×10^{-6}	3.58916×10^{-12}	3.71356×10^{-11}
0.1	7.14101×10^{-6}	1.03902×10^{-13}	5.10102×10^{-11}
0.1333	7.19259×10^{-6}	2.36748×10^{-14}	5.1737×10^{-11}

In addition, one O atom bonds to three neighboring Ti atoms, counting three Ti-O bonds, is determined by the crystalline structure of anatase TiO_2, the number of Ti-O vibration mode N can be written as:

$$N = 2n - 3nx \qquad (7),$$

where n is the number of the atoms in the TiO_2 structure, x denotes the concentration of V_O defects. Therefore, combining Eq. (2), Eq (6) and Eq. (7), I_{Raman} can be expressed as:

$$I_{\text{Raman}} \approx (2 \times 300 - 3 \times 300x)(3.57 \times 10^{-12} + \frac{5.82\times10^{-11}}{1.086}) \times (2.25714 \times 10^{-11} - 1.9577 \times 10^{-9}x + 9.65469 \times 10^{-7}x^2 - 4.904 \times 10^{-5}x^3) \qquad (8)$$

Thus, the relationship between the Raman intensity and the concentration of V_O defects is plotted in Figure 5b of main text. It can be found that the Raman intensity first decreases and then increases with decreasing the concentration of V_O defects. The calculation displays a similar variation trend consisted with the experiment data and is well explanation for experimental results (Figure 4b).

Supplementary Note 5: Raman signal of Ag-Ag interaction

To study the temperature-dependent SERS characteristics of the peaks at 88 cm^{-1} (Figure 4b), assigned to the Ag-Ag stretching vibration, the thermogravimetric and differential scanning calorimetry (TGA/DSC) of the Ag/Ag-doped TiO_2 substrate under the N_2 ambient are measured and presented in Supplementary Figure 15. According to the TGA/DSC curves, the weight loss of the as-prepared Ag/Ag-doped TiO_2 substrate is 4.93 % in the temperature range of 40-160 °C. In fact, through the analysis of experimental details, as shown in Supplementary Figure 16a, the silver citrate complex is introduced by using the trisodium citrate to reduce the $AgNO_3$ during the preparation process of the Ag/Ag-doped TiO_2 substrate. Hence, the 4.93 % weight loss resulted from the redox decomposition of silver citrate complexes in the Ag/Ag-doped TiO_2 substrate and water loss[41], which is shown in Supplementary Figure 16c. And the weight loss of the samples in the temperature range of 160 ~ 1000 °C is ascribed to the decomposition of compound 1 (Supplementary Figure 16d)[42]. These set of experimental results give us the evidence that the decrease of the signal intensities of 88 cm^{-1} in the first stage was due to the weak interactions of the Ag-Ag in the silver citrate complexes (Supplementary Figure 16b), whereas the weak interaction was broken with increasing the temperature. Furthermore, the increase of the signal intensity in the thereafter stage was ascribed to the electromagnetic enhancement of element Ag which derive from the redox decomposition of silver citrate complexes in the Ag/Ag-doped TiO_2 substrate (Supplementary Figure 16c).

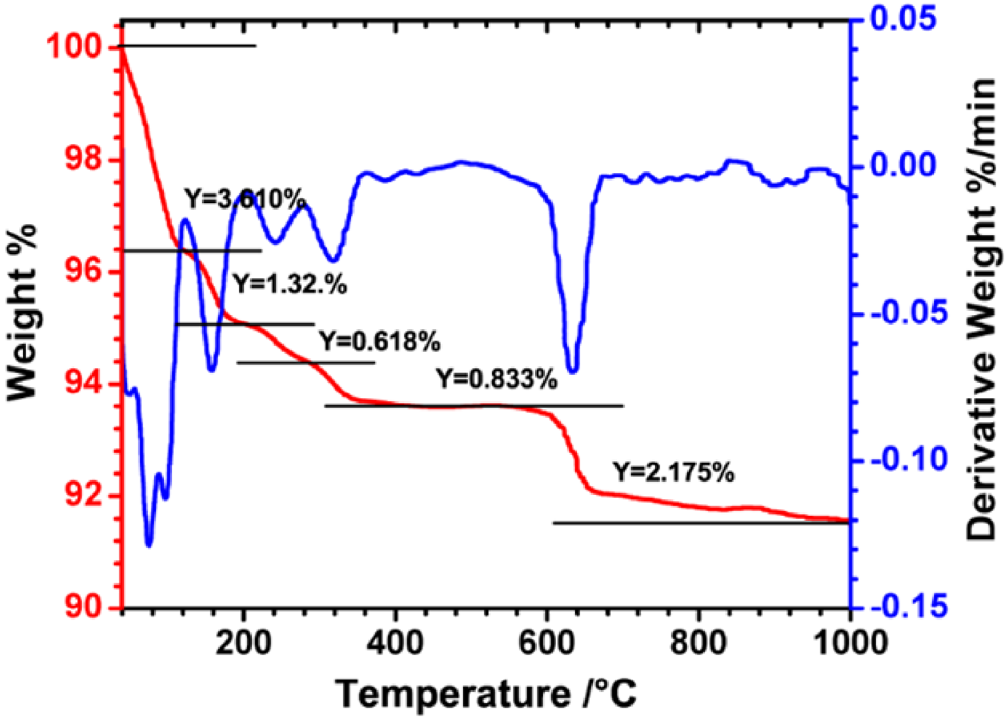

Supplementary Figure 15 Thermogravimetric and differential scanning calorimetry. TGA/DSC curve of the Ag/Ag-doped TiO_2 substrate prepared with 0.5 mM $AgNO_3$ under N_2 atmosphere.

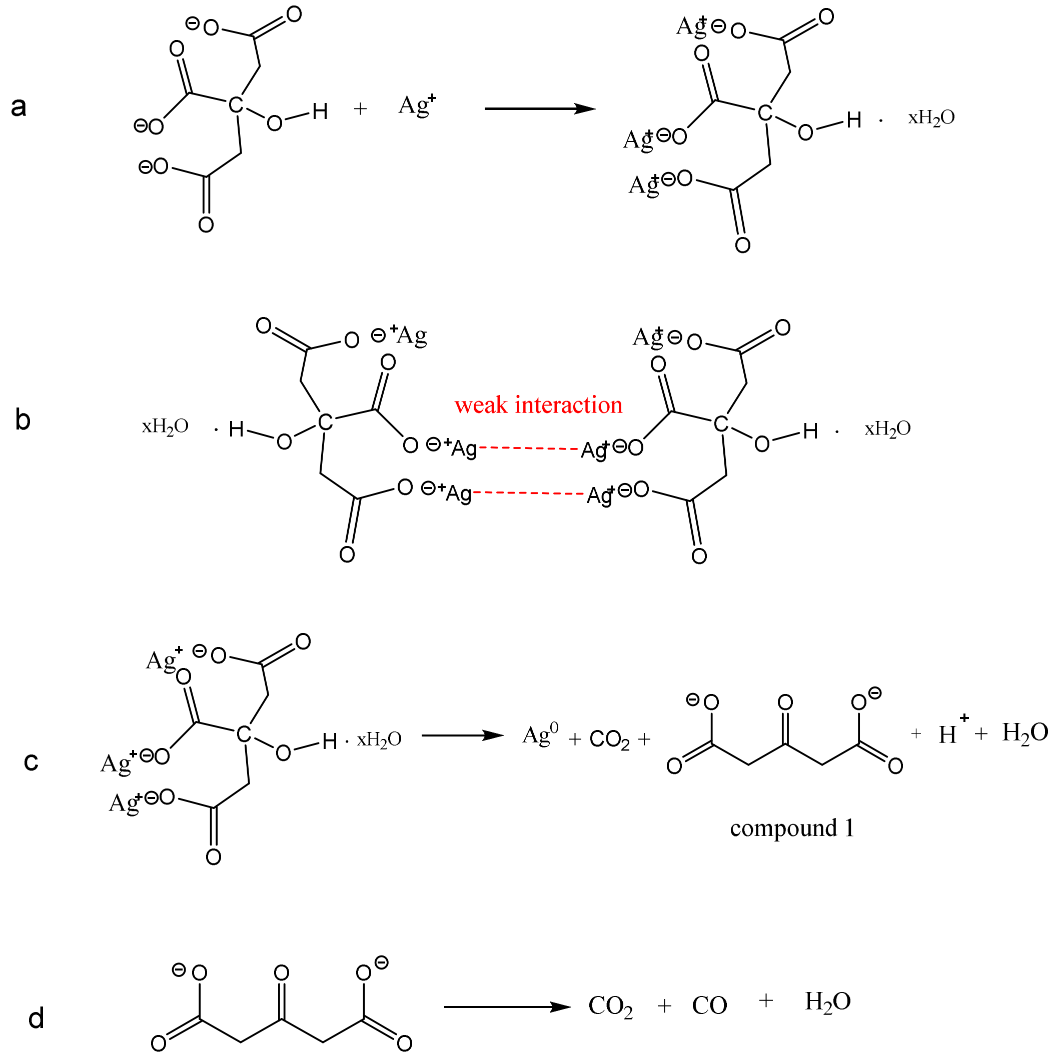

Supplementary Figure 16 Chemical reactions. (a) Formation of silver citrate complex; (b) the weak interaction of Ag-Ag in silver citrate complexes; (c) the redox decomposition of silver citrate complexes; (d) the decomposition of compound 1.

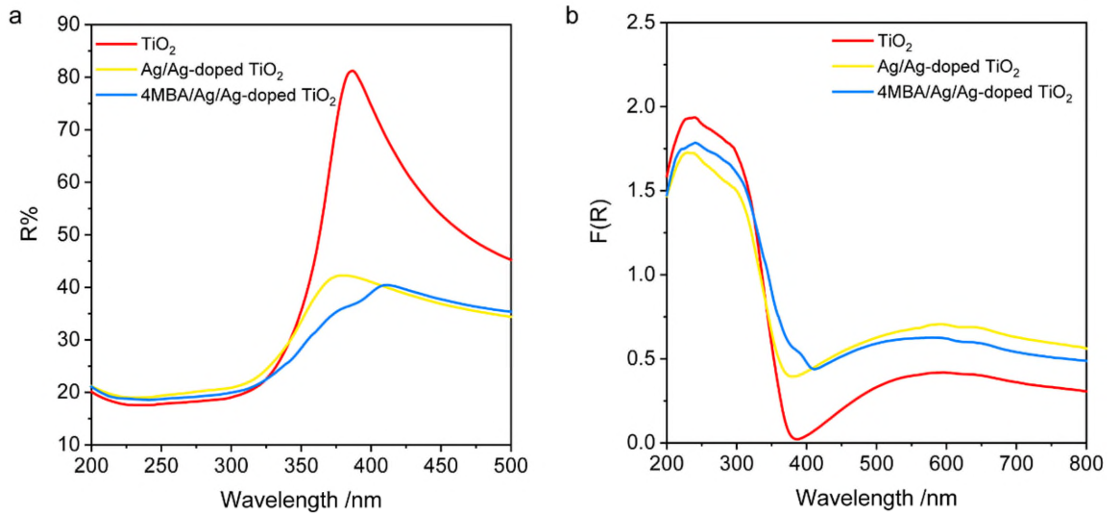

Supplementary Figure 17 UV-Vis diffuse reflection spectra. (a) Reflectance spectra and (b) Kubelka–Munk absorption curves of pure TiO_2, Ag/Ag-doped TiO_2 and 4MBA/Ag/Ag-doped TiO_2 hybrid system prepared with 0.5 mM $AgNO_3$.

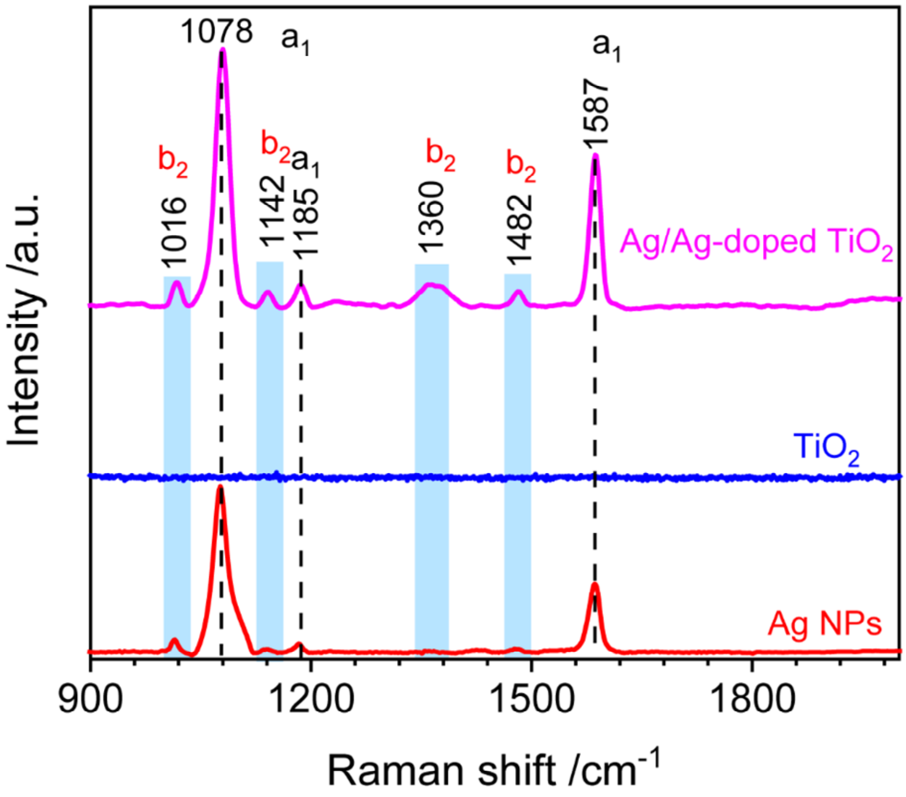

Supplementary Figure 18 CT contribution. SERS spectra of 4MBA adsorbed on Ag NPs, TiO_2 and Ag/Ag-doped TiO_2 substrate. There is no obvious SERS peak for 4MBA adsorbed on bare-TiO_2 under exciting of 785-nm laser, which is caused by the TiO_2-to-molecules CT not response.

Supplementary Table 4 Peak intensity ratio of the b_2 mode to a_1 mode

Substrate	I_{1016}/I_{1078}	I_{1142}/I_{1078}	I_{1360}/I_{1078}	I_{1482}/I_{1078}
Ag NPs	0.073	0.021	0.009	0.023
Ag/Ag-doped TiO_2	0.101	0.064	0.092	0.066

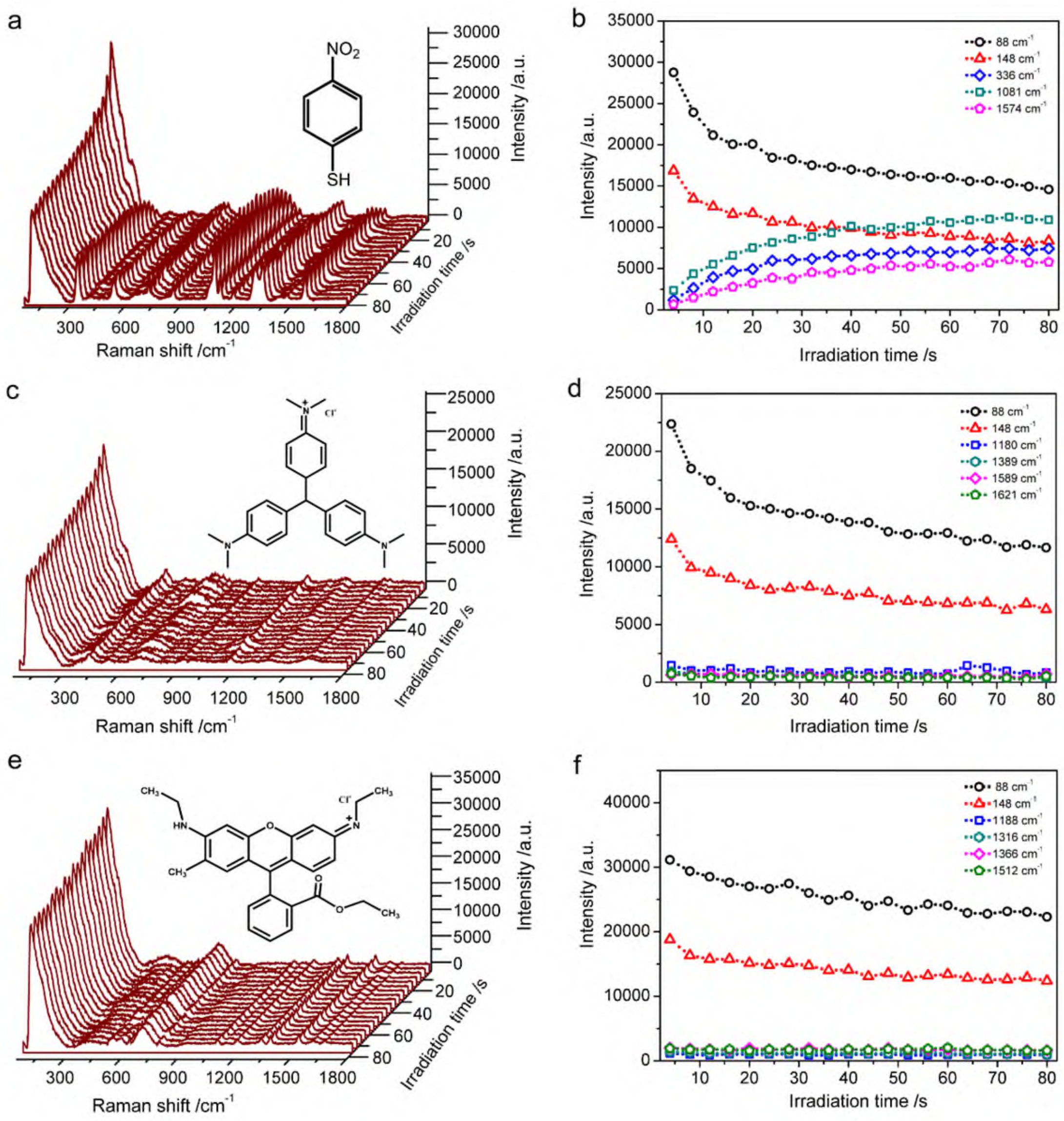

Supplementary Figure 19 SERS characteristics of molecules/Ag/Ag-doped TiO_2 hybrid system. Irradiation-time dependent SERS spectra of (a) 4NTP, (c) CV and (e) R6G adsorbed on the Ag/Ag-doped TiO_2 substrate prepared with 0.5 mM $AgNO_3$, and their temporal evolutions of SERS peak intensities (b, d and f), respectively.

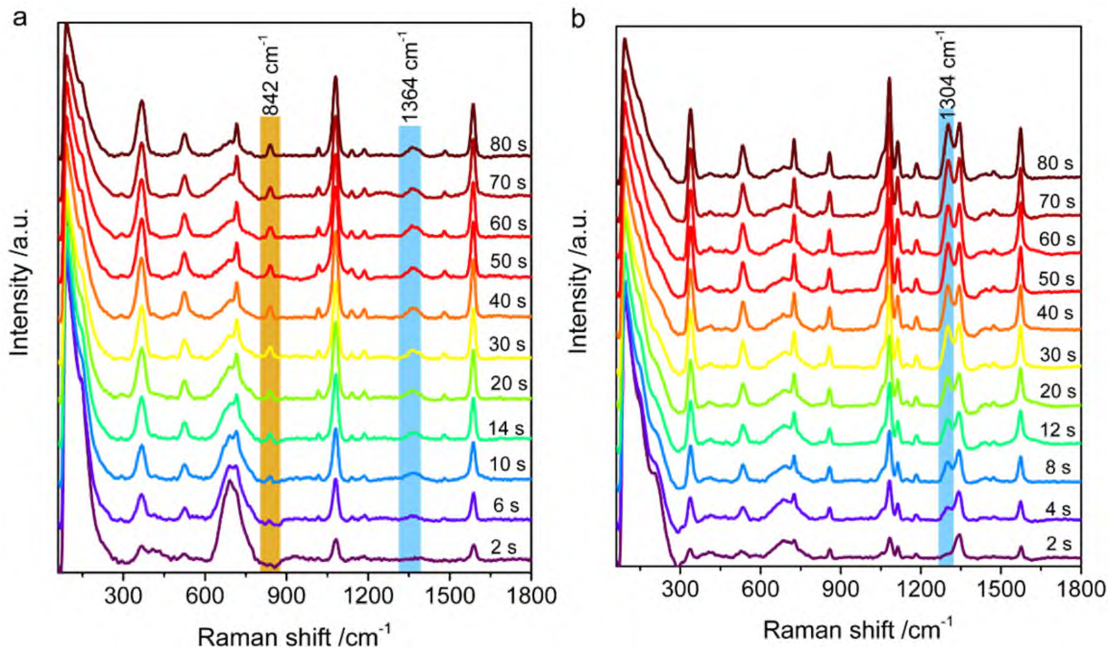

Supplementary Figure 20 Temporal evolutions of SERS spectra. Irradiation time-dependent SERS spectra of (a) 4MBA and (b) 4NTP adsorbed on the Ag/Ag-doped TiO_2 substrate prepared with 0.5 mM $AgNO_3$, respectively.

Supplementary References

1 Yang, L., Sang, Q., Du, J., Yang, M., Li, X., Shen, Y., Han, X., Jiang, X. & Zhao, B. A Ag synchronously deposited and doped TiO_2 hybrid as an ultrasensitive SERS substrate: a multifunctional platform for SERS detection and photocatalytic degradation. *Phys. Chem. Chem. Phys.* **20**, 15149-15157, (2018).

2 Zhu, X., Wang, P., Li, M., Zhang, Q., Rozhkova, E. A., Qin, X., Zhang, X., Dai, Y., Wang, Z. & Huang, B. Novel high-efficiency visible-light responsive $Ag_4(GeO_4)$ photocatalyst. *Catal. Sci. Technol.* **7**, 2318-2324, (2017).

3 Pham, T.-D. & Lee, B.-K. Effects of Ag doping on the photocatalytic disinfection of E. coli in bioaerosol by Ag–TiO_2/GF under visible light. *J. Colloid. Interf. Sci.* **428**, 24-31, (2014).

4 Chinnasamy, M. & Balasubramanian, K. Dopant induced local vibrational modes and Fano scattering in Ag doped ZnO microrods. *Spectrochim. Acta. A Mol. Biomol. Spectrosc.* **199**, 322-327, (2018).

5 He, C., Yu, Y., Hu, X. & Larbot, A. Influence of silver doping on the photocatalytic activity of titania films. *Appl. Surf. Sci.* **200**, 239-247, (2002).

6 Feng, N., Wang, Q., Zheng, A., Zhang, Z., Fan, J., Liu, S. B., Amoureux, J. P. & Deng, F. Understanding the high photocatalytic activity of (B, Ag)-codoped TiO_2 under solar-light irradiation with XPS, solid-state NMR, and DFT calculations. *J. Am. Chem. Soc.* **135**, 1607-1616, (2013).

7 Wang, S., Qian, H., Hu, Y., Dai, W., Zhong, Y., Chen, J. & Hu, X. Facile one-pot synthesis of uniform TiO2-Ag hybrid hollow spheres with enhanced photocatalytic activity. *Dalton. Trans.* **42**, 1122-1128, (2013).

8 Prakash, J., Kumar, P., Harris, R. A., Swart, C., Neethling, J. H., van Vuuren, A. J. & Swart, H. C. Synthesis, characterization and multifunctional properties of plasmonic Ag-TiO_2 nanocomposites. *Nanotechnology* **27**, 355707-355726, (2016).

9 Li, J., Xu, J., Dai, W.-L. & Fan, K. Dependence of Ag Deposition Methods on the Photocatalytic Activity and Surface State of TiO_2 with Twistlike Helix Structure. *J. Phys. Chem. C* **113**, 8343-8349, (2009).

10 Li, J. & Zeng, H. C. Hollowing Sn-Doped TiO_2 Nanospheres via Ostwald Ripening. *J. Am. Chem. Soc.* **129**, 15839-15847, (2007).

11 Xin, B., Jing, L., Ren, Z., Wang, B. & Fu, H. Effects of Simultaneously Doped and Deposited Ag on the Photocatalytic Activity and Surface States of TiO_2. *J. Phys. Chem. B* **109**, 2805-2809, (2005).

12 Mai, L., Wang, D., Zhang, S., Xie, Y., Huang, C. & Zhang, Z. Synthesis and bactericidal ability of Ag/TiO_2 composite films deposited on titanium plate. *Appl. Surf. Sci.* **257**, 974-978, (2010).

13 Chen, X. & Mao, S. S. Titanium Dioxide Nanomaterials: Synthesis, Properties, Modifications, and Applications. *Chem. Rev.* **107**, 2891-2959, (2007).

14 Yang, L., Jiang, X., Ruan, W., Yang, J., Zhao, B., Xu, W. & Lombardi, J. R. Charge-Transfer-Induced Surface-Enhanced Raman Scattering on Ag−TiO_2 Nanocomposites. *J. Phys. Chem. C* **113**, 16226-16231, (2009).

15 Wen, B. Y., Jin, X., Li, Y., Wang, Y. H., Li, C. Y., Liang, M. M., Panneerselvam, R., Xu, Q. C., Wu, D. Y., Yang, Z. L., Li, J. F. & Tian, Z. Q. Shell-isolated nanoparticle-enhanced Raman spectroscopy study of the adsorption behaviour of DNA bases on Au(111) electrode surfaces. *Analyst* **141**, 3731-3736, (2016).

16 Martin Sabanes, N., Ohto, T., Andrienko, D., Nagata, Y. & Domke, K. F. Electrochemical TERS Elucidates Potential-Induced Molecular Reorientation of Adenine/Au(111). *Angew. Chem. Int. Ed.* **56**, 9796-9801, (2017).

17 Fu, X., Bei, F., Wang, X., Yang, X. & Lu, L. Surface-enhanced Raman scattering of silylated graphite oxide sheets sandwiched between colloidal silver nanoparticles and silver piece. *J. Raman. Spectrosc.***41**, 370-373, (2010).

18 He, Y., Su, S., Xu, T., Zhong, Y., Zapien, J. A., Li, J., Fan, C. & Lee, S.-T. Silicon nanowires-based highly-efficient SERS-active platform for ultrasensitive DNA detection. *Nano Today* **6**, 122-130, (2011).

19 Wang, A.-J., Lv, J.-J., Zhou, D.-L., Weng, X., Qin, S.-F. & Feng, J.-J. Facile synthesis of ultrathin worm-like Au nanowires for highly active SERS substrates. *New J. Chem.* **38**, 3395-3400, (2014).

20 Fang, H., Zhang, C. X., Liu, L., Zhao, Y. M. & Xu, H. J. Recyclable three-dimensional Ag nanoparticle-decorated TiO_2 nanorod arrays for surface-enhanced Raman scattering. *Biosens Bioelectron* **64**, 434-441, (2015).

21 Li, X., Hu, H., Li, D., Shen, Z., Xiong, Q., Li, S. & Fan, H. J. Ordered array of gold semishells on TiO_2 spheres: an ultrasensitive and recyclable SERS substrate. *ACS Appl. Mater. Interfaces* **4**, 2180-2185, (2012).

22 Song, W., Wang, Y. & Zhao, B. Surface-Enhanced Raman Scattering of 4-Mercaptopyridine on the Surface of TiO_2 Nanofibers Coated with Ag Nanoparticles. *J. Phys. Chem. C* **111**, 12786-12791, (2007).

23 Han, D., Yao, J., Quan, Y., Gao, M. & Yang, J. Plasmon-coupled Charge Transfer in FSZA Core-shell Microspheres with High SERS Activity and Pesticide Detection. *Sci. Rep.* **9**, 13876-13881, (2019).

24 Tan, E. Z., Yin, P. G., You, T. T., Wang, H. & Guo, L. Three dimensional design of large-scale TiO(2) nanorods scaffold decorated by silver nanoparticles as SERS sensor for ultrasensitive malachite green detection. *ACS Appl. Mater. Interfaces.* **4**, 3432-3437, (2012).

25 Praveena, R., Sameera, V. S., Mohiddon, M. A. & Krishna, M. G. Surface plasmon resonance, photoluminescence and surface enhanced Raman scattering behaviour of Ag/ZnO, ZnO/Ag and ZnO/Ag/ZnO thin films. *Physica B: Condensed Matter* **555**, 118-124, (2019).

26 Chan, Y. F., Xu, H. J., Cao, L., Tang, Y., Li, D. Y. & Sun, X. M. ZnO/Si arrays decorated by Au nanoparticles for surface-enhanced Raman scattering study. *J. Appl. Phys* **111**, 033104-033109, (2012).

27 Tao, Q., Li, S., Zhang, Q. Y., Kang, D. W., Yang, J. S., Qiu, W. W. & Liu, K. Controlled growth of ZnO nanorods on textured silicon wafer and the application for highly effective and recyclable SERS substrate by decorating Ag nanoparticles. *Mater. Res. Bull.* **54**, 6-12, (2014).

28 Liu, M., Sun, L., Cheng, C., Hu, H., Shen, Z. & Fan, H. J. Highly effective SERS substrates based on an atomic-layer-deposition-tailored nanorod array scaffold. *Nanoscale* **3**, 3627-3630, (2011).

29 Quan, X., Zhao, Q., Tan, H., Sang, X., Wang, F. & Dai, Y. Comparative study of lanthanide oxide doped titanium dioxide photocatalysts prepared by coprecipitation and sol–gel process. *Mater. Chem. Phys.* **114**, 90-98, (2009).

30 Yu, J., Xiong, J., Cheng, B. & Liu, S. Fabrication and characterization of Ag–TiO_2 multiphase nanocomposite thin films with enhanced photocatalytic activity. *Appl. Catal. B Environ.* **60**, 211-221, (2005).

31 Su, C., Liu, L., Zhang, M., Zhang, Y. & Shao, C. Fabrication of Ag/TiO_2 nanoheterostructures with visible light photocatalytic function via a solvothermal approach. *CrystEngComm* **14**, 3989-3999, (2012).

32 Braun, A., Aksoy Akgul, F., Chen, Q., Erat, S., Huang, T.-W., Jabeen, N., Liu, Z., Mun, B. S., Mao, S. S. & Zhang, X. Observation of Substrate Orientation-Dependent Oxygen Defect Filling in Thin $WO_{3}-\delta$/TiO_2 Pulsed Laser-Deposited Films with in Situ XPS at High Oxygen Pressure and Temperature. *Chem. Mater.* **24**, 3473-3480, (2012).

33 Le Ru, E. & Etchegoin, P. *Principles of Surface-Enhanced Raman Spectroscopy: and related plasmonic effects*. Elsevier, Amsterdam, (2009).

34 Zhang, R., Zhang, Y., Dong, Z. C., Jiang, S., Zhang, C., Chen, L. G., Zhang, L., Liao, Y., Aizpurua, J., Luo, Y., Yang, J. L. & Hou, J. G. Chemical mapping of a single molecule by plasmon-enhanced Raman scattering. *Nature* **498**, 82-86, (2013).

35 Jiang, S., Zhang, Y., Zhang, R., Hu, C., Liao, M., Luo, Y., Yang, J., Dong, Z. & Hou, J. G. Distinguishing adjacent molecules on a surface using plasmon-enhanced Raman scattering. *Nat. Nanotechnol.* **10**, 865-869, (2015).

36 Vanderbilt, D. Soft self-consistent pseudopotentials in a generalized eigenvalue formalism. *Phys. Rev. B* **41**, 7892-7895, (1990).

37 Perdew, J. P., Burke, K. & Ernzerhof, M. Generalized gradient approximation made simple. *Phys. Rev. Lett.* **77**, 3865-3868, (1996).

38 Tobik, J. & Dal Corso, A. Electric fields with ultrasoft pseudo-potentials: applications to benzene and anthracene. *J. Chem. Phys.* **120**, 9934-9941, (2004).

39 Yu, E. K., Stewart, D. A. & Tiwari, S. Ab initiostudy of polarizability and induced charge densities in multilayer graphene films. *Phys. Rev. B* **77**, 195406-195413, (2008).

40 Li, Y., Chen, H., Huang, L. & Li, J. Ab Initio Study of the Dielectric and Electronic Properties of Multilayer GaS Films. *J. Phys. Chem. Lett.* **6**, 1059-1064, (2015).

41 Patra, S., Pandey, A. K., Sen, D., Ramagiri, S. V., Bellare, J. R., Mazumder, S. & Goswami, A. Redox decomposition of silver citrate complex in nanoscale confinement: an unusual mechanism of formation and growth of silver nanoparticles. *Langmuir* **30**, 2460-2469, (2014).

42 Rajadurai, S. Pathways for Carboxylic Acid Decomposition on Transition Metal Oxides. *Catal. Rev.* **36**, 385-403, (2006).
